# Do Illness Perceptions Predict the Attendance Rate at Diabetic Outpatient Clinic?

**DOI:** 10.5539/gjhs.v7n2p254

**Published:** 2014-11-16

**Authors:** Soontareeporn Thongsai

**Affiliations:** 1 Department of Nursing, Faculty of Nursing, Naresuan University, Phitsanulok, Thailand

**Keywords:** illness perception, non-compliance

## Abstract

**Objective:**

The aim of this study was to identify predictors of non-attendance and to investigate the influence of illness perceptions on attendance at diabetic outpatient clinics.

**Research Method and Design:**

This is a descriptive study of 442 participants who were enrolled in a Diabetic Outpatient Clinic at Lerdsin Hospital, Thailand. Illness perceptions were derived from a Thai version of the IPQ-R questionnaire consisting of the same 3 sections as the original Illness perception- Revised with the distinction that it had 68 items. Test for validity was performed and the Cronbach’s alpha reliability coefficient value was 0.87. Data were analysed by using independent t-tests. Binary logistic regression was used in order to assess the impact of illness perception scores on attendance among diabetic patients at the Outpatient Clinic.

**Results:**

The results of the study were as follows: Demographic data showed that all respondents were of Thai ethnic origin and two-thirds of the respondents were women. The average age was 60 years. The majority of the participants had completed primary school (7–12 years old), was married and were Buddhist. The IPQ-R revealed the following findings: The illness perceptions at baseline and 6 months later showed that it illness perception had no effect on the participants’ illness perception and attendance rate at the diabetic outpatient clinic. Participants who hold negative illness perceptions were more likely to attend diabetes clinics than those participants who had positive illness perceptions. A conceivable explanation for the non-significant findings in the study is the finding that during 6 month period there were some factors that have contributed to the failure of the participants to attend to their appointments. This is beyond the scope of the socio-demographic and illness perception factors.

**Conclusion:**

Thus, a plausible explanation for the non-significant findings in the study is that during 6 month period there were some factors that may have contributed to the appointment keeping among these patients beyond the scope of socio-demographic and illness perception factors.

## 1. Introduction

Patient’s views or perceptions of their illness seem to be an important variable affecting their health behaviour and problem management. Research has indicated that illness perceptions are important determinants of behaviour which are associated with adherence treatment and functional recovery (Keogh et al., 2007; Joseph et al., 2009).

In the last decade, illness perceptions have been identified as important factors that impact on the way people may change their behaviour (Petrie et al., 2007). Wilgen et al. (2008) state that “when patients are confronted with an illness or with symptoms, as in FM [fibromyalgia], they create a model and representation of this illness or symptoms (illness perceptions) in order to make sense of or try to cope with the illness and its symptoms.” This is not only true in the FM field but it has been tested in different patient groups such as MI (Myocardial Infarction), depression, anxiety disorders (Hunot et al., 2006), mild head injuries and coronary artery disease (Whittaker et al., 2007) In some processes, attributions are made in order to understand the cause of the symptoms.

In addition, research based on health psychological theories has highlighted the importance of patients’ personal beliefs about their illness and treatment in their self-management for a range of chronic illnesses (Leventhal, Meyer, & Nerenz, 1980; Petrie et al., 1996). Therefore, the way illness perception affects attendance was adopted as an important aspect of this study.

Although illness perception has been shown to be important, it is not widely used as a quantitative assessment or integrated into the clinical assessment and management of patients. In order to assess patient illness perceptions, Weinman et al. (1996) developed the Illness Perception Questionnaire (IPQ) that was later modified by Moss-Morris et al. (2002) into the revised version, IPQ-R. Since then, the IPQ-R has been used in studies of illness adaptation in patients with a wide range of conditions, including heart disease (Cooper et al., 1999), rheumatoid arthritis (Murphy et al., 1999), chronic fatigue syndrome (Heijmans, 1999) and diabetes (Griva et al., 2000), paediatric (Janse et al., 2005) and chronic illness (Keith et al., 2007).

### 1.1 The purpose of the Research

1). To identify predictors of non-attendance
2). To investigate that those participants who hold positive illness perceptions are more likely to attend diabetes clinics than those participants who have negative illness perceptions at 24 weeks after baseline.

### 1.2 Research Theoretical Framework

In addition to consideration of theoretical dimensions of the way reminders may work, the *theory of planned behaviour* has been noted as useful explanatory theory that is likely to assist in gaining a better understanding of this phenomenon, and in designing a more robust intervention, and more rigorous and appropriate means of its evaluation.

One of the strengths of the TPB is that it not only identifies the main factors associated with behaviours, but it also addresses beliefs that underpin these determinants, namely behavioural, normative and control beliefs. Within the model, it is argued that attitudes stem from expectations about the results of performing specific activities (behavioural beliefs), weighted by the value placed on these outcomes (outcome evaluations). Similarly, perceptions about what others perceive as being important can affect behaviour (normative beliefs), and this is weighted by motivation to comply with these views (motivation to comply). Finally, TPB is underpinned by beliefs about factors that facilitate or inhibit behaviour (control beliefs), weighted by the expected impact of these factors if they are present (perceived power). We might assume that a benefit of the belief-based model is to use it to identify those beliefs that may influence behaviour and thus to provide avenues for interventions designed to change such behaviour.

It is important to distinguish between failure to adhere because of memory lapses or problems understanding the regimen (Weinstaub, 1976) and individual beliefs about illness course and importance of particular aspects of management. Such factors may affect diabetes clinic attendance behaviour and may therefore be considered as potential confounders in evaluations. Therefore, the present study also examined that the positive illness perception may predict the attendance rate for diabetic outpatient clinic for patient with type 2 diabetes.

### 1.3 The Scope of the Research

A descriptive research recruited participants with type 2 diabetes who attending the diabetic outpatient clinic at Lerdsin Hospital on the study period time. Finally, there were 442 participants enrolled in the study.

### 1.4 Participants

Participants were patients who required routine follow-up appointment at a diabetic outpatient clinic at Lerdsin Hospital. Consent was sought from all patients (total sample size required) who fulfilled the following criteria:

#### 1.4.1 Inclusion Criteria

- Patients who are diagnosed with type 2 diabetes (ICD-10) using the International Statistic Classification of Disease.
- Age over 18 years (the legal age for informed consent in Thailand)
- Attend a follow up appointment at the diabetes outpatient clinic during the study period.
- Patients who scheduled their appointment more than one working day before their appointment
- Informed consent from the patient or a relative of the patient

#### 1.4.2 Exclusion Criteria

- Patients who did not attend follow-up appointment at the diabetes outpatient clinic at Lerdsin Hospital, Thailand during the study period and those whose appointment was scheduled less than one working day before their appointment
- Did not sign the informed consent form

## 2. Research Methodology

### 2.1 Research Design

Description of Instrument

The IPQ-Revised (IPQ-R) was used as a tool for this study to measure participant’s illness perception regarding diabetic disease. It originally consisted of 3 sections comprised of 70 items. For the purposes of this study, the original IPQ-R was translated into the Thai. Terminology was altered for clarification where appropriate; for example, the term “my illness” was changed to “my diabetes”. It is important to note 2 phrases in part 2 (VIEW ABOUT YOUR DIABETES) of the original IPQ-R could not be translated (i.e. “My diabetes makes me feel angry” and “My diabetes does not worry me”). Thus, with the permission of the IPQ-R author, the researcher excluded both from the final translated version. The adapted questionnaire was entitled “IPQ-R-DM (Thai language version)” and consists of the same 3 sections as the original IPQ-R with the distinction that it was made up of 68 items in accordance with the above-mentioned translational restrictions. It has been test for validity and The Cronbach’s alpha reliability coefficient values were 0.87

### 2.2 Ethical Considerations

1). Permission to recruit for this study was sought and obtained from the director of the Lerdsin hospital.
2). All staff involved were informed of the study
3). Patient consent forms were obtained.

### 2.3 Procedure for Data Collection

After obtaining ethics approval from the Lerdsin Hospital some modifications to improve clarity of the information sheet and consent form and feasibility of the proposed timetable were made in accordance with recommendations of the review committee. Individual consent was required for this study, and a letter of approval was presented to diabetic outpatient clinic, in which the study took place. Informed consent was obtained on the same day that the participants were recruited. Participants were informed that their participation in the study was voluntary and they could refuse to answer any specific questions or they could terminate their participation at any point any time during the study period without giving reason.

## 3. Results of the Study

### 3.1 Demographic Information

Characteristics of the sample, including age, gender, education, marital status, religion, occupation, medical support, accommodation, number of family members and duration of (DM) illness, were measured. All patients were Thai ethnic origin and two-thirds were female (see Table 1). The average age of participants in the study was 58 years. The majority of the participants had completed primary school (7–12 years old), were married and Buddhist. Details of particular occupation were limited, but ‘housewife/house husband’ was the largest category chosen amongst those recorded, followed by self-employed. Interestingly, the majority of patients reported receiving health benefit from the local direct payment scheme (30 Baht Project). Around half of the participants owned their accommodation. No significant differences were found in demographic characteristics between the patients participating in the 24 weeks assessment and those who were lost to follow-up.

**Table 1:** The table shows the demographic characteristics of patients (n= 442)

**Variables**		Number	Percentage
***Age***	Aged 58 or below	232	(52.48%)
	Aged above 58 years	210	(47.52%)
***Gender***	Male	140	(31.67 %)
	Female	302	(68.32%)
***Education***	Primary school (age7-12 years)	235	(53.10%)
	Higher than primary (13-18 years)	201	(45.47%)
***Marital status***	Single	68	(31.00%)
	Married	370	(83.71%)
***Religion***	Buddhist	372	(84.16%)
	Other	64	(14.47%)
***Occupation***	Housewife/Husband	170	(38.46%)
	Self-employment	175	(39.59%)
***Health benefit***	Local direct payment scheme (30 Baht)	191	(43.21%)
	State health plan	103	(23.30%)
***Accommodation***	Owner	225	(50.90%)
	Other	210	(47.51%)
***Family size***	Less than, or equal to, 4 people	261	(59.04%)
	More than 5 people	181	(40.96%)

### 3.2 Illness Perception (Patient Experiences and Knowledge of Their Diabetes Mellitus)

Data were collected about patient experiences and their knowledge of diabetes mellitus symptoms. In this section, the question contained two parts asking about “Your Views about Diabetes”. As exhibited in Table 2, the percentage of participants scoring above the scale midpoint provides an indication of the proportion of participants endorsing stronger than the average beliefs about each construction. Of the common 14 experiences stated in the IPQ-R the majority of participants experienced loss of strength, fatigue and dizziness.

**Table 2:** The table shows the patient experiences and knowledge of diabetes mellitus

Symptoms	I have experienced these symptoms *since my diabetes* n= 442		This symptom is *related to my diabetes* n=442	

	Number	%	Number	%
1. Pain	196	44.3	136	30.8
2. Sore throat	111	25.1	70	15.8
3. Nausea	122	27.6	98	22.2
4. Breathlessness	90	20.4	71	16.1
5. Weight loss	210	47.5	197	44.6
6. Fatigue	255	57.7	237	53.6
7. Stiff joints	114	25.8	93	21.0
8. Sore eyes	157	35.5	153	34.6
9. Wheeziness	62	14.0	56	12.7
10. Headaches	148	33.5	103	23.3
11. Upset stomach	105	23.8	68	15.4
12. Sleep difficulties	204	46.2	129	29.2
13. Dizziness	235	53.2	197	44.6
14. Loss of strength	275	62.2	247	55.9

**Table 2:** The table shows the patient experiences and knowledge of diabetes mellitus (continue)

VIEW ABOUT YOUR DIABETES	Baseline Mean (SD)	Exit point Mean (SD)
	Mean (SD)	Mean (SD)
***Section 2***		
1. My diabetes will last a short time	3.89 (1.05)	3.90 (1.19)
2. My diabetes is likely to be permanent rather than temporary	3.86 (1.01)	3.79 (1.02)
3. My diabetes will last for a long time	3.92 (0.96)	3.94 (0.95)
4. This diabetes will pass quickly	3.55 (1.07)	3.48 (1.25)
5. I expect to have this diabetes for the rest of my life	3.94 (1.06)	3.82 (1.17)
6. My diabetes is a serious condition	2.51 (1.03)	2.52 (1.12)
7. My diabetes has major consequences on my life	2.81 (1.16)	2.92 (1.28)
8. My diabetes does not have much effect on my life	2.72 (1.18)	2.78 (1.24)
9. My diabetes strongly affects the way others see me	2.49 (0.93)	2.41 (0.89)
10. My diabetes has serious financial consequences	2.42 (1.16)	2.39 (1.11)
11. My diabetes causes difficulties for those who are close to me	2.27 (1.07)	2.34 (1.03)
12. There is a lot which I can do to control my symptoms	3.60 (0.99)	3.70 (0.95)
13. What I do can determine whether my diabetes gets better or worse	3.55 (0.86)	3.72 (0.83)
14. The course of my diabetes depends on me	3.66 (0.94)	3.63 (1.02)
15. Nothing I do will affect my diabetes	3.52 (1.08)	3.67 (1.12)
16. I have the power to influence my diabetes	3.50 (0.94)	3.54 (0.93)
17. My actions will have no affect on the outcome of my diabetes	3.59 (0.95)	3.72 (1.08)
18. My diabetes will improve in time	2.78 (1.14)	3.05 (1.29)
19. There is very little that can be done to improve my diabetes	3.30 (1.09)	3.38 (1.21)
20. My treatment will be effective in curing my diabetes	3.90 (0.87)	3.78 (0.99)
21. The negative effects from my diabetes can be prevented (avoided) by my treatment	3.72 (0.88)	3.61 (1.01)
22. My treatment can control my diabetes	3.96 (0.88)	3.89 (0.86)
23. There is nothing which can help my condition	3.46 (1.00)	3.59 (1.11)
24. The symptoms of my condition are puzzling to me	3.42 (0.90)	3.38 (1.12)
25. My diabetes is a mystery to me	3.23 (1.07)	3.45 (1.16)
26. I don’t understand my diabetes	3.54 (1.03)	3.68 (1.17)
27. My diabetes does not make any sense to me	3.59 (1.00)	3.61 (1.14)
28. I have a clear picture or understanding of my condition	3.71 (0.93)	3.58 (0.97)
29. The symptoms of my diabetes change a great deal from day to day	2.38 (1.04)	2.42 (1.04)
30. My symptoms come and go in cycles	3.12 (1.06)	3.01 (1.06)
31. My diabetes is very unpredictable	2.98 (1.06)	2.89 (1.07)
32. I go through cycles in which my diabetes gets better or worse	2.98 (1.19)	2.94 (1.13)
33. I get depressed when I think about my diabetes	2.48 (1.24)	2.39 (1.13)
34. When I think about my diabetes I get upset	2.48 (1.19)	2.56 (1.23)
35. Having this diabetes makes me feel anxious	2.49 (1.25)	2.48 (1.19)
36. My diabetes makes me feel afraid	2.52 (1.27)	2.50 (1.20)

**Table 2:** The table shows the patient experiences and knowledge of diabetes mellitus (continue)

POSSIBLE CAUSES OF DIABETES	Baseline Mean (SD)	Exit point Mean (SD)
	Mean (SD)	Mean (SD)
***Section 3***		
1. Stress or worry	2.49 (0.95)	2.64 (1.02)
2. Hereditary it runs in my family	3.19 (1.20)	3.21 (1.17)
3. A germ or virus	2.49 (1.32)	2.67 (1.29)
4. Diet or eating habits	3.25 (1.21)	3.14 (1.20)
5. Chance or bad luck	2.58 (1.06)	2.66 (1.07)
6. Poor medical care in my past	2.86 (1.02)	2.78 (1.05)
7. Pollution in the environment	3.04 (1.09)	2.95 (1.15)
8. My own behaviour	3.29 (1.14)	3.29 (1.23)
9. My mental attitude, e.g. thinking about life negatively	2.95 (1.05)	3.05 (1.13)
10. Family problems or worries	3.00 (1.16)	2.98 (1.14)
11. Overwork	3.09 (1.17)	3.00 (1.18)
12. My emotional state, e.g. feeling down, lonely, anxious, empty	2.97 (1.13)	3.11 (1.12)
13. Ageing	3.12 (1.20)	3.01 (1.11)
14. Alcohol	3.02 (1.12)	3.10 (1.22)
15. Smoking	3.33 (1.21)	3.30 (1.21)
16. Accident or injury	2.74 (1.30)	2.84 (1.29)
17. My personality	3.46 (1.24)	3.37 (1.29)
18. Altered immunity	3.11 (1.22)	3.14 (1.21)

The three main symptoms that participants believed were associated with the illness were also loss of strength, fatigue and dizziness. Whereas, the other symptoms such as pain, sore throat, nausea, breathlessness, weight loss, stiff joints, sore eyes, wheezing, headache, upset stomach, and sleep difficulties were endorsed with a lower number. It is possible that participants were under the assumption that these symptoms did not affect their diabetes.

#### 3.2.1 Patient Experiences and Knowledge of Their Diabetes Mellitus

The results on the outcome measures at baseline that the results showing across participants who responded about their ***own personal views of now how do you see your current diabetes*** indicated that at baseline the top 5 categories were: “(22). My treatment can control my diabetes”, “(5). I expect to have this diabetes for the rest of my life”, ‘(3). My diabetes will last for a long time”, “(20). My treatment will be effective in curing my diabetes” and “(1). My diabetes will last a short time”.

Values are indicated according to participant’s agreement or disagreement with the statement ratingfrom 1 = strongly disagree to 5 = strongly agree (Likert’s scale)

Illness perceptions about the ***possible cause of diabetes*** are described that the majority of participants endorsed those causes they believed were related to their diabetes. At baseline the most important items were (17). “My personality”, (15). “Smoking”(8). “My own behavior”, (4). “Diet or eating habits” and (2) “Hereditary - it runs in my family”.

Four of the 18 items on the IPQ-R indicated that participants were more likely to believe in internal causes for the possible causes of diabetes, including “My own behavior, personality and smoking habit”. Participants were more likely to think that their diabetes was related to poor diet and eating habits However, at two different point of time did not differ in their duration of illness timeline (acute/chronic), timeline (cyclical), beliefs about personal control.

Psychological and risk factors may be related to an increased sense of personal and treatment control. Participants endorsed high scores on behavioural and psychological casual factors such as smoking and “my own behaviour”. On the other hand, immune attributions, which incorporated external causal factors, including heredity, accident and chance, contrast highly to treatment control, timeline and cyclical timeline. Participants who made more psychological attributions also had a tendency to view their illness as chronic and were more distressed by their illness.

A lack of association between illness perceptions and outpatient attendance was evident (Table 3). Participants who held negative perceptions were slightly more likely to attend than those whose illness beliefs were positive: 42% of participants who held negative illness perception attended their baseline appointment compared to 36% of those with positive illness perception. A chi-square test of the relation between baseline attendance and positive or negative illness perception was not significant (P = 0.380).

**Table 3:** The table shows the relationship between illness perceptions and attendance at baseline

Scale	Appointment attendance at the baseline point		
Illness perception	Did not attend N (%)	Attend N (%)	Total N (%)
***Negative illness perception***	121 (58.2%)	87 (41.8%)	208 (100%)
***Positive illness perception***	126 (63.6%)	72 (36.4%)	198 (100%)

There were some missing values; data were presented according to number of participants who had completed the revised illness perception questionnaires.

### 3.3 Binary Logistic Regression of Baseline Attendance

A binary logistic regression model was used to further examine the relationship between baseline attendance and demographic variables: age, gender, marital status, religion family size, duration of illness (DM), timeline (chronic/acute), personal control and illness coherence.

Results of these analyses (Table 5) show that four of the independent variables had significant partial effects. The odds ratio for duration of illness, timeline (chronic/acute), personal control and illness coherence indicates that when holding all other variables constant, participants who believe that their illness is chronic are less likely to attend the appointment than participants who believe their illness is acute. Furthermore, participants who have been diagnosed with type 2 diabetes for less than 9 years, have understanding about their disease and have strong beliefs that their illness can be controlled or cured are more likely to attend their appointment rather than those who did not. The variation explained was 22.7% (Nagelkerke R^2^=0.227*100).

**Table 5:** The summary of binary logistic regression analysis to predict appointment attendance rate

Characteristics	B	S.E.	Wald	Sig	Exp(B)	95% C.I. for	
						Lower	Upper
***Age***	0.008	0.008	0.924	0.337	1.008	0.992	1.024
***Gender***	-0.151	0.243	0.388	0.533	0.860	0.534	1.383
***Marital status***	0.099	0.317	0.097	0.756	1.104	0.593	2.053
***Religion***	-0.438	0.306	2.046	0.153	0.646	0.354	1.176
***Family size***	0.243	0.232	1.103	0.294	1.275	0.810	2.008
***Duration of illness***	0.991	0.243	16.569	‹0.001	2.693	1.67	4.338
***Timeline (acute/chronic)***	-1.022	0.199	26.381	‹0.001	0.360	0.244	0.532
***Personal control***	0.850	0.236	12.950	0.001	2.340	1.473	3.718
***Illness coherence***	0.458	0.215	4.520	0.034	1.580	1.036	2.409

Nagelkerke R^2^=0 227; Log Likelihood=484.296; Chi-Square=7.213; Sig=0.514.

Dependent variables entered: Exit point appointment. Variables entered: participants’ group, baseline appointment, timeline (acute/chronic), personal control and illness coherence.

### 3.4 Relationship Between Exit Point Attendances, Patients’ Characteristics and Their Illness Perceptions

The final model shown in Table 6 revealed that the addition of demographic information factors and illness beliefs were not significantly (P≤0.05) associated with attendance at diabetic outpatient clinic for type 2 diabetic patients at their exit point appointment. The odds ratio for the group variable was 0.977, again not much different from the odds ratio of 0.896 when only the group variable is included. Demographic information and illness perception dimensions did not contribute as predictors of appointment keeping for patients with type 2 diabetes. The variation of the change that may have influenced the attendance rate was calculated to be 8.2 % (Nagelkerke R^2^=0.082*100).

**Table 6:** The table showed the summary of binary logistic regression analysis to predict appointment attendance rate

Characteristics	B	S.E.	Wald	Sig	Exp(B)	95% C.I. for	
						Lower	Upper
***Baseline appointment***	-0.709	0.390	3.296	0.069	0.492	0.229	1.058
***Age***	0.002	0.013	0.020	0.888	1.002	0.976	1.028
***Gender***	0.224	0.392	0.327	0.568	1.251	0.580	2.700
***Marital status***	-0.368	0.503	0.535	0.465	0.692	0.259	1.854
***Religion***	-0.794	0.635	1.564	0.211	0.452	0.130	1.569
***Family size***	0.616	0.360	2.933	0.087	1.851	0.915	3.745
***Duration of illness***	0.413	0.384	1.162	0.281	1.512	0.713	3.207
***Timeline (acute/chronic)***	0.297	0.285	1.089	0.297	1.346	0.770	2.354
***Personal control***	-0.401	0.356	1.270	0.260	0.670	0.333	1.345
***Illness coherence***	-0.423	0.350	1.458	0.227	0.655	0.330	1.302

Nagelkerke R^2^= 0.082-2; Log Likelihood=238.930; Chi-Square=8.398; Sig=0.396.

## 4. Discussion

Based on the results of the study, the IPQ-R showed that illness perception had no effect on the paticipants’ attendance or compliance to attend to appointments in diabetic clinics. These findings are inconsistent with the results of similar studies previously published, including three diabetes studies and two myocardial infarction studies (Barnes, Moss-Morris, & Kaufusi 2004; Hjelm et al. 1999; Hjelm et al. 2005). These contradictory findings are surprising, given the non-significant association in attendance rate at 24 weeks found in the study. The IPQ-R is a measure of patients’ beliefs about what caused their illness, how long it will last, its expected controllability, and the emotional reaction to their illness (Ellzabeth et al., 2011; Horne et al., 2002; Pimm, & Weinman, 1998; Hejjmans, 1998b; Moss-Morris et al., 1996; Petrie et al., 1996). The responses on this test are, therefore, considered predictive of the medication adherence and clinic attendance. In general, the results showed that mean scores of illness perception for participants in different point of time were alike. It may be concluded that illness perceptions did not affect the attendance rate in the study. Furthermore, the attendance rate at the baseline appointments showed that participants who had negative illness perception were more likely to attend their appointments than participants who held positive illness perceptions. It is possible that participants who held negative illness perception had a less comprehensive understanding about the nature their illness and felt less confident to confront their illness. This assumption is supported by the study of Barnes et al. (2004) and John et al. (2007), in which patients who understood the necessity for medications were more likely to adhere to their medication regimen. On the other hand, at the exit point appointment the results of the illness perception showed that the association between participant’s illness perception and their attendance at diabetic outpatient clinic had slightly changed but was not significant. At this stage (exit point), participants who held negative illness perception were less likely to present at their appointment compared with those who held positive illness perception. Regarding the timeline (acute/chronic), at baseline, participants were largely perceived that their diabetes was chronic in nature. They also believed that the treatment could control their illness. These could be the other factors that influenced the participants to attend their final appointment (exit point). This statement was supported by the study by Lai et al. in which patients’ ideas of illness course and perceived severity of the disease as a whole were assumed to be associated with their adherent behaviours (Lai, Chie, & Lew-Ting 2007; Day, 1995). Thus, a plausible explanation for the non-significant findings in the study is that during a 6 month period there were some factors that may have contributed to the appointment keeping among these patients beyond the scope of socio-demographic and illness perception factors.

## 5. Implications for Nursing Research, Practice, and Education

The results of this study indicate that patients with type 2 diabetes who have either a positive illness perception or a negative illness perception could benefit and attend their appointment. Hence, it would be desirable to further assess the factors that are influential to participants’ attendance to their appointment regardless of their illness perceptions.
